# Hive Transplantation Has Minimal Impact on the Core Gut Microbiome of the Australian Stingless Bee, *Tetragonula carbonaria*

**DOI:** 10.1007/s00248-023-02222-w

**Published:** 2023-04-24

**Authors:** T. J. T. Mills, T. M. Nelson, L. A. Pearson, B. A. Neilan

**Affiliations:** School of Environmental and Life Sciences, The University of Newcastle, Callaghan, New South Wales Australia

**Keywords:** Lactobacillaceae, Symbiosis, Corbiculate, Meliponiculture, Metabolic function

## Abstract

Bacteria residing in the guts of pollinating insects play a key role in nutrient acquisition, digestion, and resistance to pests and diseases. Imbalances in microbial flora in response to environmental change and stress can therefore impact insect health and resilience. This study is aimed at defining the core gut microbiome of the Australian native stingless bee, *Tetragonula carbonaria*, and exploring the impact of colony transplantation on gut health. The gut microbiomes of nine forager bees from natural (log) and manufactured (box) hives were examined via 16S rRNA gene amplicon sequencing. Some differences were observed at the ASV level between the microbiomes of log and box hive bees. However, a core microbiome, dominated by *Lactobacillus* spp., unclassified Acetobacteraceae spp., and *Bombella* spp., was maintained. Further, the inferred functional potential of the microbiomes was consistent across all individuals. This study highlights that although hive transplantation has an impact on the overall diversity of stingless bee gut microbiomes, it is unlikely to have a significant negative impact on the overall health and resilience of the colony.

**Supplementary Information:**

The online version contains supplementary material available at 10.1007/s00248-023-02222-w.

## Introduction

The Australian stingless bee, *Tetragonula carbonaria* (Hymenoptera: Apidae), is found along the eastern coast of Australia from Queensland to New South Wales. It is one of the few Australian corbiculate (pollen basket) bee species to form large social colonies and plays a vital role in the pollination of native and commercially relevant plants [1]. Like many other ecologically and economically important pollinating insects, stingless bees in Australia are threatened by human activities and environmental factors, including pesticide use, land clearing, the introduction of exotic plant and pest species, bushfires, and extreme weather conditions [2–4]. To preserve and engage with these industrious insects, an increasing number of amateur apiarists have taken up stingless beekeeping (meliponiculture) [5, 6].

Stingless beehives can be rescued from the environment when destructive actions such as tree clearing reveal their existence. In such cases, the hives can either remain in their existing cavity, for example, within a cut log or tree branch, or they may be transplanted into an artificial cavity within a specifically manufactured wooden box [7]. Manufactured hives are also used to propagate stingless bee colonies by splitting an existing hive into two boxes when capacity within the first hive is limited. Australian stingless beekeepers have increased 2.5-fold in the last decade, and the number of colonies sold each year has more than quadrupled, placing increased reliance on box hives [6]. While meliponiculture is an important strategy for preserving both pollinator and plant biodiversity, it is unclear what impacts hive transplantation has on the health and resilience of stingless bees.

Microbial associations in the insect gut have been shown to be fundamentally important to the development and vitality of insects (Sabree, Kambhampati, and Moran 2009; Evans and Armstrong 2006; Yoshiyama and Kimura 2009). These alliances can provide nutritional and reproductive advantages, as well as defense against parasites and disease [8, 9]. The insect gut microbiome can therefore provide insight into the overall health and resilience of the host.

Previous studies of corbiculate bees, including the Western honeybee, *Apis mellifera*, Eastern honeybee, *A. cerana*, and bumblebees, *Bombus* spp., showed that their gut microbiomes are strongly conserved, despite their broad geographical distribution [3, 10–13]. A study that looked at individuals across three major clades of bees, representing 25 species, revealed that *Snodgrassella*, *Gilliamella*, *Bifidobacterium*, and *Lactobacillus* spp. are prevalent in the gut microbiome and make up the core corbiculate gut microbiome [10]. An *Acetobacter*-like taxon was unique to the group of stingless bees that includes *T. carbonaria* [10]. Despite the presence of a core microbiome in corbiculate bees, different host species and individuals maintain unique microbial associations due to differences in available food resources, host ecology, and physiology. This phenomenon is more pronounced in *T. carbonaria* [3, 10].

In line with their highly conserved core microbiome, corbiculate bee guts also support broadly conserved microbial metabolic functions related to digestion of pollen and nectar and resistance to pests and diseases [14–18]. For example, functions associated with carbohydrate metabolism and sugar uptake reflect adaptations to the host’s diet, which is rich in sugars [19]. Gut microorganisms also contribute to the metabolism of lipids and proteins, the detoxification of plant secondary metabolites and pesticides [20, 21], and the production of antimicrobial compounds [22].

Considering the above, studying bee gut microbiomes can potentially inform nutritional and defense strategies in different bee populations and provide a snapshot of their overall health. This study is aimed at defining the core gut microbiome of *T. carbonaria* and exploring the impact of colony transplantation on gut health. Specifically, we wanted to know (i) whether gut microbiome taxonomic diversity differed between individual foragers from natural (log) and manufactured (box) hives and (ii) whether gut microbiome functional potential differed between individual foragers grouped by hive type. The results are discussed with respect to the conservation of these intriguing eusocial insects.

## Methods

### Sample Collection

*Tetragonula carbonaria* forager bees were collected from two hives located in Hatton Vale, QLD, Australia, latitude 27° 33′ 5.3″ S, longitude 152° 29′ 56″ E. The first hive, denoted as “log hive,” housed a naturally established colony located within a hollow log. The second hive denoted “box hive” housed a transplanted colony located within a wooden box. Hives were located approximately 65 m apart to minimize the risk of drift foragers between the hives. Twelve forager bees were collected from each hive by placing a sterile 50 mL tube over the entrance of the hive and trapping the bees as they exited. The bees were immediately frozen in dry ice and stored at -80 °C prior to processing.

### DNA Extraction

Thawed bees were surface sterilized in 1 mL of 70% v/v aqueous ethanol with sonication for 45 s at 60% power level using a Benchtop Ultrasonic Cleaner 250TD (Soniclean, Australia) and then rinsed in 1 mL 0.154 M NaCl solution. The whole gut was removed using a sterile scalpel blade and forceps and placed in a sterile 1.5 mL tube.

Bacterial genomic DNA was extracted from individual bee guts using a modified version of the XS method [23]. One mL of XS buffer (1% w/v potassium ethyl xanthogenate, 800 mM NH4OAc, 100 mM Tris–HCl (pH 7.4), 20 mM EDTA, and 1% w/v sodium dodecyl sulfate (SDS)) was added to cryo-vials containing silica beads and gut samples. Samples were lysed in a Fast Prep 120 (Savant Instruments) bead-beater for 45 s at 6 beats/s followed by incubation at 65 °C for 2 h. Lysed cell suspensions were chilled on ice for 10 min and centrifuged at 12,000* g* for 10 min. The resulting supernatants were transferred to sterile tubes containing 1 mL phenol:chloroform:isoamyl alcohol (25:24:1). Samples were mixed via inversion and centrifuged at 12,000 g for 10 min at 4 °C. The upper aqueous layer was transferred to fresh tubes and 2 volumes of 100% ethanol, and 0.1 volumes of 3 M sodium acetate were added. The tubes were incubated overnight at -20 °C and precipitated DNA was collected via centrifugation at 14,000 g for 20 min at 4 °C. Following the removal of supernatants, DNA pellets were washed with 70% ethanol and centrifuged at 14,000 g for 10 min. Washed DNA pellets were air dried then resuspended in 20 μL of sterile milli-Q water. DNA yield and quality were checked using a Nanodrop® spectroscopy system (Thermo Scientific), and final DNA concentrations were adjusted to ~ 30 ng/μL^−1^ with sterile Milli-Q water.

### 16S rRNA Gene Pyrosequencing

The V1-V3 variable region of the bacterial 16S rRNA gene was amplified from the extracted DNA by PCR using the HotStarTaq Plus Master Mix Kit (Qiagen, USA) and the primers 27F and 519R [24]. Reaction conditions were as follows: an initial denaturation step (94 °C for 3 min) followed by 35 cycles of denaturation (94 °C for 30 s), annealing (53 °C for 40 s), and extension (72 °C for 90 s), and then a final elongation step (72 °C for 5 min). The resulting 500 bp PCR amplicons were sequenced at the Research and Testing Laboratory (Lubbock, Texas) using a Roche GS FLX Titanium instrument. Sequence data are available on the National Center for Biotechnology Information Sequence Read Archive under BioProject accession number PRJNA940519.

### Bioinformatics

Raw sequences were converted into an Amplicon Sequence Variant (ASV) abundance table using QIIME2 (Quantitative Insights Into Microbial Ecology) version 202.8.0 [25] implemented in Python version 3.6.11. Demultiplexed sequence counts from 12 samples ranged between 5,888 and 34,471 (Table S1). The DADA2 pipeline [26], implemented in QIIME2, was used to filter and trim the first 20 bases from each read and truncate sequences to 425 bases. The remaining sequences were dereplicated, chimeras were removed, and finally, ASVs were generated for downstream analysis. After quality filtering, there were 98 unique ASVs. To account for the fact that not all species are equally likely to be observed in the bee intestinal habitat type, taxonomic assignment of ASVs was performed using the trained and weighted Silva 99% full length sequence database version 138 [27, 28] with the q2-feature-classifier plugin [29]. Further identification of taxa was obtained through BLAST (Basic Local Alignment Search Tool) via the National Center for Biotechnology Information interface (blast.ncbi.nlm.nih.gov/Blast.cgi), and results were reported with consideration of the amplicon similarity, coverage, and the number of base hits expected to be seen by chance (*E* value).

Of the 98 taxa, all were assigned to the kingdom Bacteria with the exception of sample box-4 which showed > 85% of reads were unclassified at the level of kingdom (Figure S1) and therefore this sample was removed from further analysis. Data were additionally pruned to remove representatives classified to chloroplast (*n* = 1) and mitochondria (*n* = 1) at the taxonomic level of family. The count range of the remaining samples was from 2,680 to 8,652 reads per sample with an average count per sample of 4,770.9 (Table S1). The remaining dataset had 89 taxa from 29,601 counts across eleven samples. ASV filtering and pruning was conducted with the package *phyloseq* version 1.42.0 [30] in the R statistical program version 4.2.1 [31].

### Statistical Analyses

Rarefaction curves (Figure S2) did not identify significant loss for samples with lower counts and showed a plateau of the curve to indicate samples representing the diversity that is present in the bee gut. As a result, all remaining samples were kept for downstream analysis. Alpha diversity metrics including observed, Shannon’s [32] and Chao [33] were estimated based on ASVs and visualized with the *phyloseq* package version 1.42.0 in R statistical software version 4.2.1 (Figure S3). The core microbiome was defined as those ASVs identified in six or more of the eleven *T. carbonaria* individuals. A heat map of the ASVs in the core microbiome was generated and visualized using the “plot_core” function from the *microbiome* package version 1.19.1 [34] in R version 4.2.1. To identify beta diversity across samples, weighted and unweighted UniFrac distance matrices were visualized on principal coordinate analysis plot following rarefaction of all samples to even depth in the package *phyloseq* version 1.42.0. Differences in the whole and core bee microbiome as measured by UniFrac were assessed by conducting a permutational multivariate analysis of variance (PERMANOVA) using the command “adonis2” in the package *vegan* version 2.6.4 [35] in R version 4.2.1 with the grouping factor hive.

Functional pathways were inferred from 16S rRNA gene amplicon data using the tool, phylogenetic investigation of communities by reconstruction of unobserved states (PICRUSt2 version 2.5.0) [36]. Sample, box-4, was removed from downstream analysis to mimic the 16S rRNA dataset. The functional pathway unstratified abundance data table identified 311 pathways from 11 samples. Data were pruned to remove features present in only 30% of individuals, and pathways described as “super pathways” or “engineered” were excluded from analyses [36]. The final data set included 211 functional pathways. Abundance counts were rounded to the nearest whole number using the function “round” in base R version 4.2.1. *ALDEx2* package version 1.30.0 [37] was run in R version 4.2.1 with default options to identify features that differed significantly between bees in different hive types. Results for all statistical tests were considered significant where *p*-values < 0.05.

## Results

**Fig. 1:**
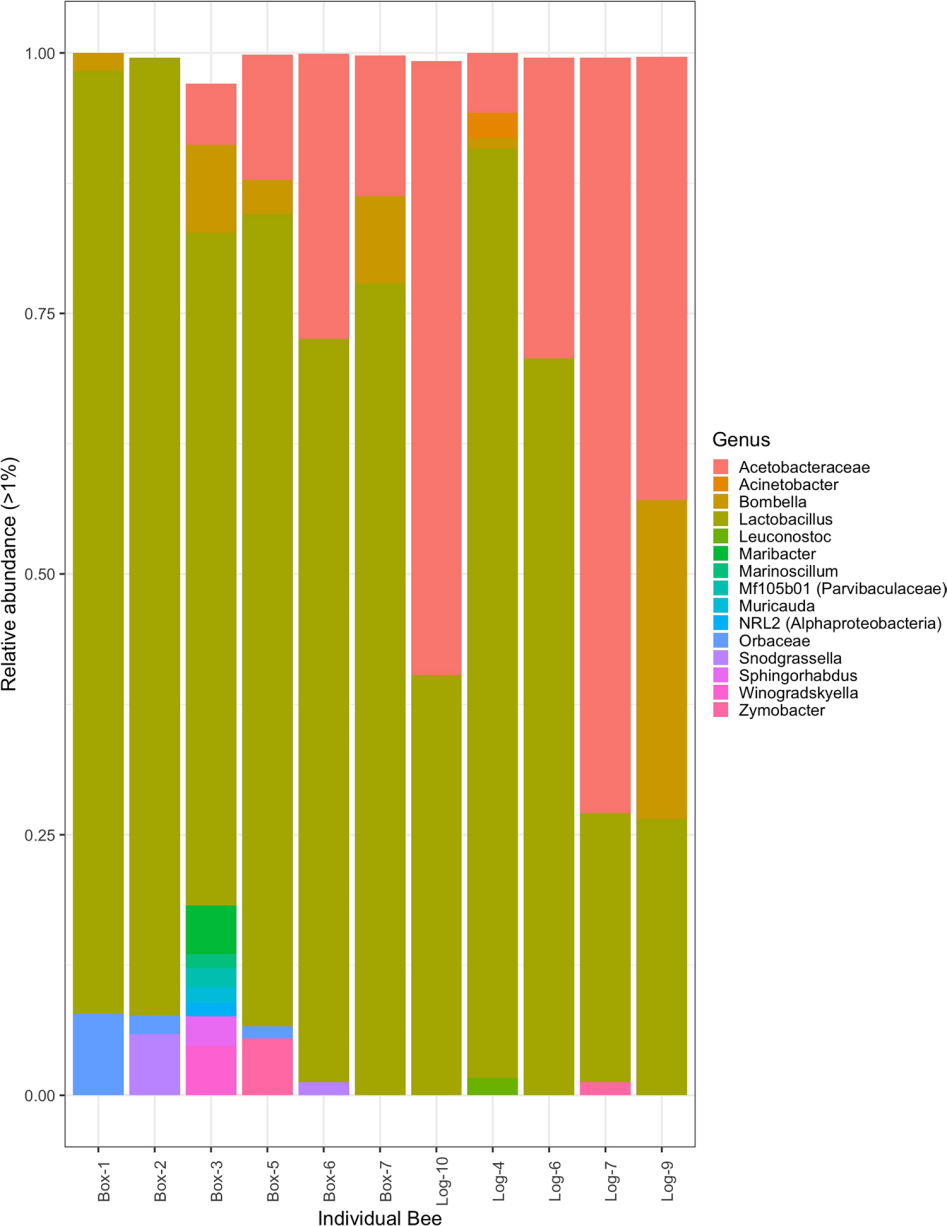
Relative abundance of bacterial genera in the bee gut. Bar plot shows the relative abundance of ASVs greater than 1% found in the gut of 11 T*. carbonaria* individuals from two hive types: artificial, “box” and natural, “log”. To account for differences in sampling depth between samples, data were rarefied prior to conversion to relative abundance. Colors indicate the summed relative abundance at the level of genus in an individual bee sample

Amplifiable bacterial DNA was obtained from the whole dissected gut of five bees taken from log hives and six bees taken from box hives. Following quality filtering, 16S rRNA gene pyrosequencing yielded 52,480 sequence reads which were classified into 84 distinct ASVs. There were 77 ASVs from 37 and 14 genera with greater than 1% abundance counts in any individual sample (Fig. 1). Three ASVs represented more than 50% in any one sample; in all cases, they were classified to the genus *Lactobacillus* (Table S2). While *Lactobacillus* was the most dominant taxon overall (65% of all ASVs), Acetobacteraceae (25%) and *Bombella* (5.15%) were also abundant (Table S3). Individual bees averaged 11 unique ASVs in their gut with the observed number of species ranging from 4 to 11 for all but one individual, box-3, which had 40 ASVs (Figure S3). We investigated the presence of common bee or insect pathogens in samples, including *Melissococcus*, *Paenibacillus*, *Lysinibacillus*, *Serratia*, or *Spiroplasma* spp. but no ASVs representing these bacteria were observed.

**Fig. 2:**
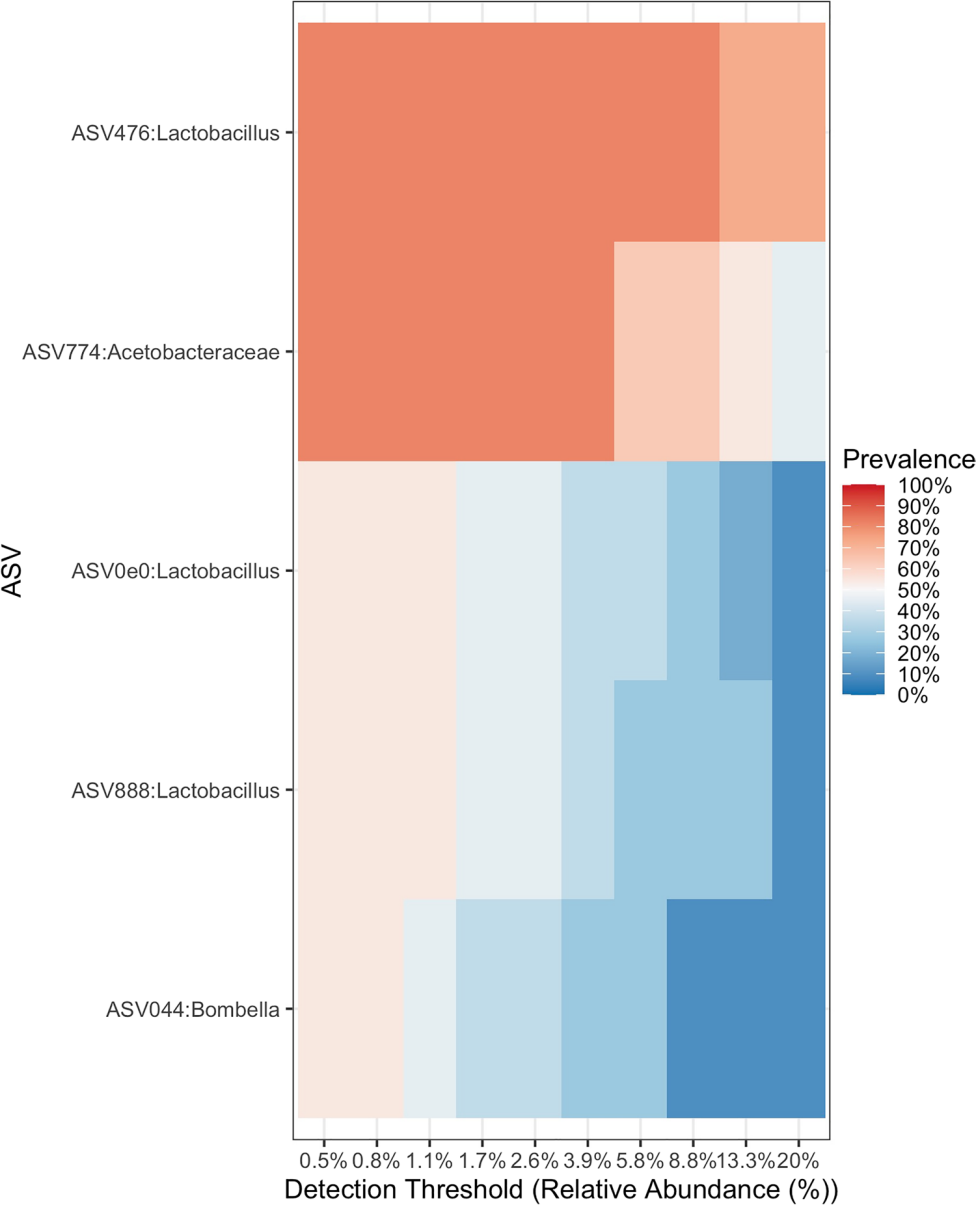
Relative abundance and prevalence of ASVs in the *T. carbonaria* core gut microbiome. Core microbiome was defined as ASVs identified in six or more of the eleven *T. carbonaria* individuals. Relative abundance refers to the average abundance of a bacterial amplicon sequence variant (ASV) across all bees. Prevalence refers to the total number of individual bees from which the ASV was identified divided by eleven (and expressed as a percentage). For example, a prevalence of 100% indicates that an ASV was identified in eleven out of eleven bees; a prevalence of 50% indicates that an ASV was identified in six out of eleven bees

The core *T. carbonaria* gut microbiome, defined as ASVs present in at least 50% of the individuals, consisted of five unique ASVs from three taxonomic classifications (Fig. 2). *Lactobacillus* was the most prevalent genus across bees represented by three unique ASVs. The most prevalent ASV, identified in all but one individual and classified to the genus *Lactobacillus*, was identical (*E* value = 0) in a BLAST search to *Bombilactobacillus thymidiniphilus* (Table 1). The second most prevalent ASV, classified to the family Acetobacteraceae, showed the greatest similarity (*E* value = 2.00 -170) in a BLAST search to the species, *Neokomagataea tanensis*. The ASV classified to the genus *Bombella* and identified in five of the six box hive individuals and one log hive individual was identical in a BLAST search to the species, *Bombella mellum* (Table 1). The two remaining ASVs present in more than 50% of individuals were similar to the species *Lactobacillus crispatus* (Table 1 and Fig. 2). We identified the occurrence of previously identified core bee gut ASVs classified to *Gilliamella*, *Snodgrassella*, and *Zymobacter* spp. in just a few individual bees but not more than 50% of individuals in the dataset.

**Table 1:** Core ASVs identified with BLAST search

Core ASVs						
Lowest taxonomic classification	Prevalence (%)	Scientific name	Query cover^**^ (%)	*E* value^^^	Identity^^^^ (%)	Accession^^*^
*Lactobacillus* (ASV476)	90.9	*Bombilactobacillus thymidiniphilus*	100	0	99.8	OM986476.1
Acetobacteraceae (ASV774)	81.8	*Neokomagataea tanensis*	100	2 x 10^-170^	92.5	CP032485.1
*Bombella* (ASV044)	63.6	*Bombella mellum*	100	0	96.9	MT787561.1
*Lactobacillus* (ASV888)	54.5	*Lactobacillus crispatus*	100	2 x 10^-164^	91.6	LC065039.1
*Lactobacillus* (ASV0e0)	54.5	*Lactobacillus crispatus*	100	2 x 10^-164^	91.6	LC065039.1

Core microbiome was defined as ASVs (amplicon sequence variants) identified in six or more of the eleven *T. carbonaria* individuals. Prevalence refers to the total number of individual bees from which the ASV was identified divided by eleven (and expressed as a percentage). Basic local alignment search tool (BLAST) was used to identify similarity to known available sequences in the National Center for Biotechnology Information (NCBI) database. Searches were conducted on the 5th of October 2022. ^**^Query coverage: the % of the contig length that aligns with the NCBI hit. ^^^*E* value: the number of hits expected to be seen by chance. ^^^^Percent identity: the % of bases that are identical to the reference genome. ^^*^Accession: identification number that links this sequence submission in NCBI

Alpha diversity did not indicate any differences between the mean species diversity within the gut microbiomes of bees from box and log hives (Figure S3). However, the whole gut microbiomes of log and box hive bees were distinct (PERMANOVA: weighted and unweighted UniFrac < 0.05, Table 2). On visual inspection, the individual bees from the distinct hives clustered together in the PCoA beta-diversity plots except for sample, box-3 (Figure S4). Further investigations into what taxonomic groups are driving the differences between box and log hive bees did not yield any statistically significant differences. However, trends were observed with an increase in the presence of *Lactobacillus* spp. and a decrease in Acetobacteraceae spp. (Table S3) in box hive bees when compared to log hive bees (Figure S5).

**Table 2:** Difference between bee gut microbiomes grouped by hive type

Grouping	DF	Sum of squares	*F*	*P*
Hive type: whole microbiome (weighted UniFrac)	1	0.14565	5.0231	0.0245^*^
Hive type: whole microbiome (unweighted UniFrac)	1	0.40789	2.2399	0.0046^**^
Hive type: core microbiome (weighted UniFrac)	1	0.06544	0.613	0.4671
Hive type: core microbiome (unweighted UniFrac)	1	0.05009	1.5702	0.2372

PERMANOVA conducted on rarefied ASV abundances from the whole and core microbiome converted to unweighted and weighted UniFrac distance matrices with 9,999 permutations. Core microbiome was defined as ASVs with a presence in more than 50% of individual bee gut samples and resulted in a total of five taxa. All permutations tested non-significant for homogenous multivariate dispersion when tested, where ^*^p value = 0.01–0.05, ^**^p value = 0.0001–0.01. Plot of data points are displayed in principal coordinates analysis (Figure S4)

**Fig. 3:**
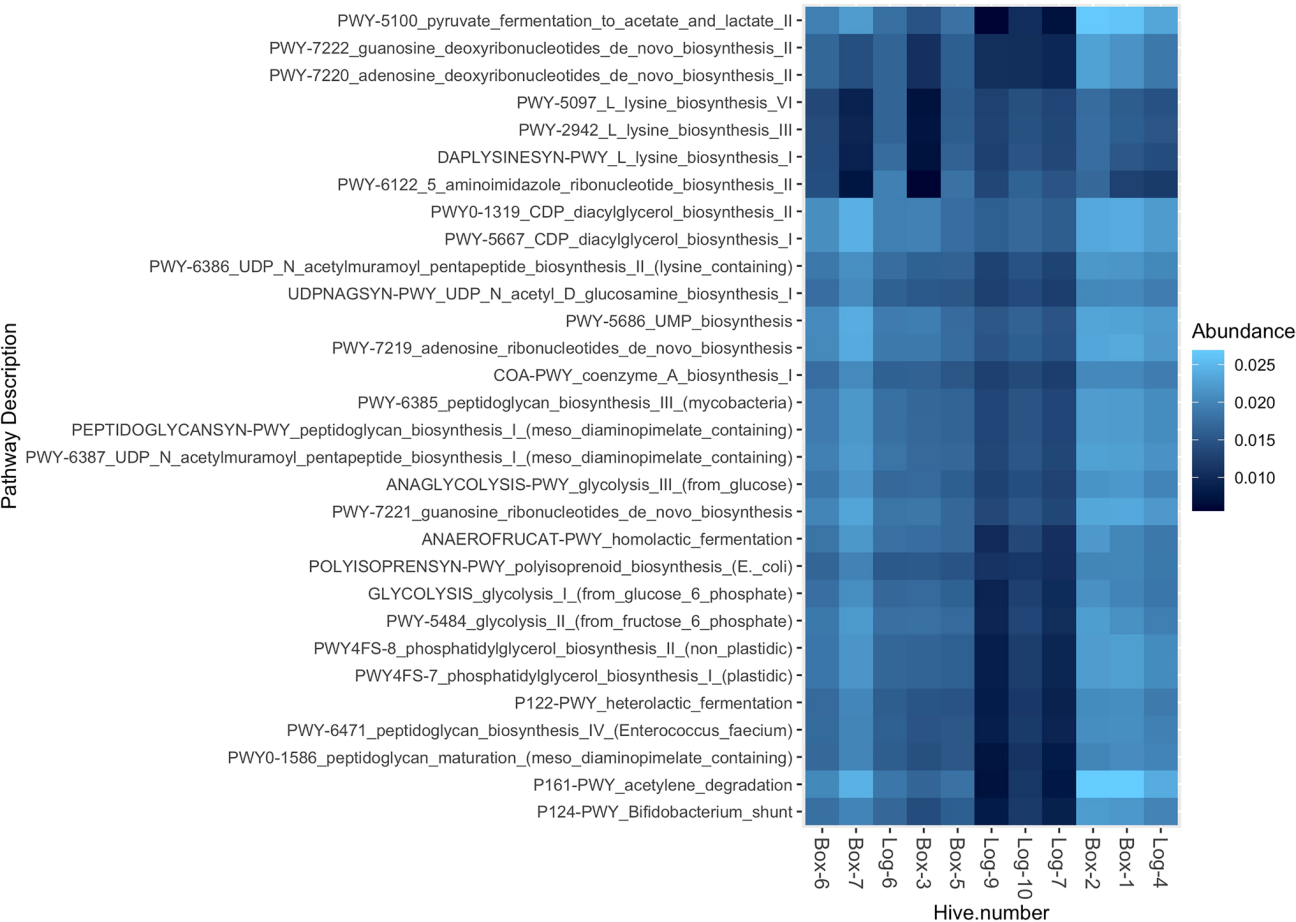
Top 30 functional pathways present in all individual bee gut microbiomes. Functional pathways inferred from 16S rRNA gene amplicon sequencing indicates over 122 pathways present in 80% of individual bees (full list in Table S5). Heat map displays the top 30 functional pathways present in all individual bees

Functional pathways were inferred from 16S rRNA gene amplicon sequences and indicated there were 169 functional pathways present in the gut microbiomes of all 11 bee individuals. From these functional pathways, 122 were present in 80% of all bees and 108 were present in all bees sampled. The top functions identified with the greatest abundance across individual bees included pathways involved in diacylglycerol and phosphatidylglycerol (phospholipid) biosynthesis, pyrimidine, and purine (DNA and RNA precursor) biosynthesis, peptidoglycan and acetylmuramoyl (cell wall component) biosynthesis, glycolysis, lysine (amino acid) biosynthesis, and pyruvate fermentation (Fig. 3). Differential testing did not identify significant differences in pathways between bees grouped by hive type (Table S6).

## Discussion

Microorganisms colonizing the gut are contributors to, and indicators of, insect health. This is particularly true for bees, which rely on specific microbial taxa for the digestion of pollen and carbohydrates, detoxification of sugars and harmful chemicals, and resistance to pests and diseases [38–42]. This study aimed to define the core gut microbiome of the Australian stingless bee, *T. carbonaria*, from a pilot cohort of bees from two hives and exploring the impact of colony transplantation on gut health.

The natural and log hive bees shared a core microbiome of five taxa represented by *Lactobacillus*, Acetobacteraceae, and *Bombella*, consistent with observations in the microbiome of other corbiculate bee species, including stingless bees [3, 10, 43]. Two of these ASVs were identical to *Bombilactobacillus thymidiniphilus* and *Bombella mellum*, isolated from *T. carbonaria* sampled from Brisbane, Australia, and the honeycombs of the Western honeybee, *Apis mellifera*, respectively [44, 45]. Previous studies have demonstrated that *Lactobacillus* spp. are dominant members of the honeybee microbiome and have strong and long (> 80 mya) evolutionary ties to their host [19, 21, 22, 46]. The abundance and diversity of *Lactobacillus* spp. in the *T. carbonaria* gut microbiome and the occurrence of other core ASVs isolated from related habitats suggests taxa exist in the gut that have co-evolved with the host and play key roles in this bee species. In this study, hives were approximately 65 m apart from one another, suggesting individual bees did not drift between the hives. However, interactions between individuals at local foraging resources or from mixing with unsampled hives located in closer proximity (approximately 25 m) cannot be completely ruled out.

Although not represented in the core, the genera *Gilliamella*, *Snodgrassella*, and *Zymobacter* were present in more than one individual, consistent with previous studies [3, 10]. *G. apicola*, *S. alvi*, and *Z. palmae* are members of the class Proteobacteria and have been observed in other bee hosts or hives. *G. apicola* and *S. alvi* are consistently reported in *A. mellifera* and *Bombus* spp. hosts where *Z. palmae* and *Zymobacter* spp. have been observed in bee hosts to a lesser extent and more transiently [3, 10, 43, 47, 48]. The reduced occurrence of these species in the gut of *T. carbonaria*, compared to other bee hosts, may signal their role is varied and dependent on available niches in the microbiome of each individual.

Previous studies suggest that bacteria occupying the bee gut microbiome assist with the digestion and preservation of food [49], protect bees by outcompeting or killing harmful microorganisms [39], and increase the fitness of their hosts by priming their immune systems [38–40]. In line with previous metagenomic studies in *A. mellifera*, we identified pathways broadly related to intestinal homeostasis and health, carbohydrate metabolism, and amino acid biosynthesis [17, 19, 22]. Pathways involving the conversion of pyruvate into lactate and pyruvate into acetate (the foundations of lactic and acetic acid fermentation, respectively) were abundant. Several of the taxa identified in this study are capable of this conversion, including *Lactobacillus*, *Zymobacter*, or Acetobacteraceae spp. Lactobacilli metabolize glucose to form lactic acid and members of the Acetobacteraceae metabolize ethanol to acetic acid [50]. Kešnerová et al. (2017) showed that individual bees mono-colonized with *G. apicola* accumulated pyruvate, whereas *S. alvi* and *Lactobacillus* spp. (Firm-5) utilized this substrate, suggesting taxa in this habitat may have syntrophic interactions. The production of acids shape the physicochemical environment of the intestines by lowering the pH and oxygen levels in the gut with implications for host health (Zheng et al. 2017). For example, the reduced pH is thought to enhance resistance to the intestinal parasite, *Crithidia bombi* in the bumblebee, and *Bombus terrestris* [39] and has been shown to inhibit spoilage microbes and other pathogens [51, 52] and exclude the growth of the opportunistic insect pathogen, *Serratia marcescens* in the honeybee gut [53]. Our results suggest that the presence of similar functional activities of the core microbiome may influence the host in beneficial ways.

Acetobacteraceae is a common component of bee gut microbiomes [54, 55], and there is some evidence that these are particularly prevalent in stingless bees [10, 56]. In the honey bee, members of the family Acetobacteraceae, including *Bombus* spp., occupy defined niches within the hive including queen guts, nurse crops, nurse glands, and the royal jelly and are not prevalent in the gut across all individuals [49, 57, 58]. One dominant member of the Acetobacteraceae, *Bombella mellum*, in *T. carbonaria* was identified in seven of the eleven bee individuals. The Acetobacteraceae is common in sugar-loaded environments where they commonly perform oxidative fermentation, converting sugars into alcohol and acetic acid. We observed a high abundance of fermentation pathways in the gut of all bee individuals, such as ubiquinol and glycerol biosynthesis, glycolysis, and other sugar degradations. The high abundance of Acetobacteraceae and *Bombella* spp. in the *T. carbonaria* gut microbiome, not exclusive to the queen but across all individuals, suggests they play a unique role in this host. *T. carbonaria* honey has a unique abundance of the naturally occurring isomer of sucrose and trehalulose [59], where honey from *A. mellifera* and *A. florea* is dominated by the monosaccharides, fructose, and glucose, with unreported traces of trehalulose [60]. This distinctive sugar composition of honey and the prevalence of *Bombella* and Acetobacteraceae spp. in the gut of *T. carbonaria* could indicate a role in metabolic homeostasis unique to this host.

Interestingly, *Bifidobacterium* spp., a dominant component of the core gut microbiome in honeybees (*Apis* spp.) and bumble bees (*Bombus* spp.) [10, 19, 61], was absent from our cohort. *Bifidobacterium* spp. degrades polysaccharides such as hemicellulose and pectin and plays a significant role in harvesting nutrients [62] and modulating host behavior [20]. While this taxon has been observed in previous studies of stingless bee microbiomes, it tends to occur in low abundance and does not form part of the “core” microbiome [3, 43, 63]. It is possible that *Lactobacillus* spp. fulfills this role in *T. carbonaria* as it has been shown in experiments on mono-colonized honey bees that individual strains can take up the bulk of metabolic output if required, and *Lactobacillus* spp. is particularly adept at this [64].

Previous studies have shown that host taxonomy, available botanical resources, and exposure to pesticides are the main drivers of bee gut diversity [3, 65–67]. Even identical species from close locations are known to have variations in their abundances of microbes. Our study cohort was from the same local population and was exposed to similar environmental forces, which explains the presence and similarity of the core microbiome and inferred metabolic functions of the bee gut microbiomes. However, differences between log and box hives, equating to different chemistry (treated versus untreated timber and tannins), humidity, temperature, and airflow, could explain the distinct whole microbiomes in log and box hive bees. Alternatively, the stress of colony transplantation could make individuals more susceptible to collecting novel bacteria from their surroundings, as has been observed previously for honey bees [68]. While hive transplantation is a disruptive process, we found only moderate evidence of gut flora imbalance in the box hive bees. For example, the box hive bees had an overabundance of *Lactobacillus* spp*.* and a reduced abundance of *Acetobacteraceae* spp., compared to the log hive bees. Due to the small number of sampled individuals, these trends may be more pronounced in larger studies. Importantly, we did not identify any bacterial genera or species with known links to disease manifestation (e.g., *Melissococcus*, *Paenibacillus*, *Lysinibacillus*, *Serratia*, or *Spiroplasma* spp.) [69] in either hive, suggesting that our cohort was pathogen free. Outwardly, these hives were thriving, and it is therefore likely that the differences in the whole microbiome were superficial, and the presence of a shared core and functional profiles further supports this theory.

Consistent with other studies of corbiculate species, the overall alpha diversity in the gut was low with an average of eight unique ASVs in an individual, from a total of 77 ASVs observed across all bee individuals. Kwong et al. (2017) observed from 1 to 22 unique operational taxonomic units (OTUs) in each individual and a total of just 199 OTUs in their study which sampled 472 individuals representing 27 bee species [10]. In our dataset, there was one exceptional individual, box-3, which had 40 unique ASVs present in its gut microbiome. This individual also had the highest read depth of all bees, and hence, data analysis methods were chosen with the ability to control for this unevenness. The observed differences at the ASV level between individuals may reflect recent exposure to different floral resources or pesticides, resulting in transient changes in gut flora. However, in line with previous studies, the presence of the core microbiome in our study cohort (including box-3) clearly demonstrates that host-mediated forces stabilize bee gut microbiota over longer timescales [10, 19, 47].

The functional pathways identified in the gut environment of *T. carbonaria* indicate their role in provision and removal of metabolites and compounds for the host. Synthesis and provisioning of amino acids by gut microorganisms for their animal hosts is known to occur [70]. Here, we inferred functional pathways for the biosynthesis of most amino acids in the gut microbiome including lysine, isoleucine, and tryptophan, which are considered essential in honeybees [71]. This is in support of other studies that have observed amino acid biosynthesis as part of the role of the bee gut microbiome. In a study of the genomes on 231 isolates from honeybee gut bacteria, Zheng et al. (2019) identified the presence and absence of genes upregulated for amino acid biosynthesis. There is evidence to suggest the production and synthesis of certain amino acids by the gut microbiota, specifically strains of *Lactobacillus* spp., invoke host brain gene expression which impacts neurological behavior and memory [15]. Pollen and nectar often contain amino acids, but not always, and the functional capacity of the *T. carbonaria* gut microbiota to synthesize most amino acids provides the flexible option, if necessary, to synthesize those that are not available directly from the diet.

This study has shown that *T. carbonaria* hosts a core microbiome of functionally important bacteria, including *Lactobacillus* spp. and Acetobacteraceae spp. which are important for nutrient acquisition, defense, and host homeostasis. Our inferred functional pathway analysis suggests that while hive transplantation impacts the whole microbiome, the core microbiome (and its functional potential) is preserved. These results provide a positive outlook for the Australian bee-keeping industry, which is heavily reliant on hive splitting and transplantation for the preservation and propagation of colonies.

## Supplementary Information

Below is the link to the electronic supplementary material.

Supplementary file1 (XLSX 62.9 KB)

Supplementary file2 (JPG 419 KB)

Supplementary file3 (JPEG 211 KB)

Supplementary file4 (JPG 238 KB)

Supplementary file5 (JPG 283 KB)

Supplementary file6 (JPEG 389 KB)

## Data Availability

The sequence data generated during this study will be made available on the National Center for Biotechnology Information Sequence Read Archive under BioProject accession number PRJNA940519.
