# Supplementary figures and images for: Hive Transplantation Has Minimal Impact on the Core Gut Microbiome of the Australian Stingless Bee, Tetragonula carbonaria
Source: Microb Ecol. 2023 Apr 24;86(3):2086–96. doi: 10.1007/s00248-023-02222-w (PMC10497653; doi:10.1007/s00248-023-02222-w)

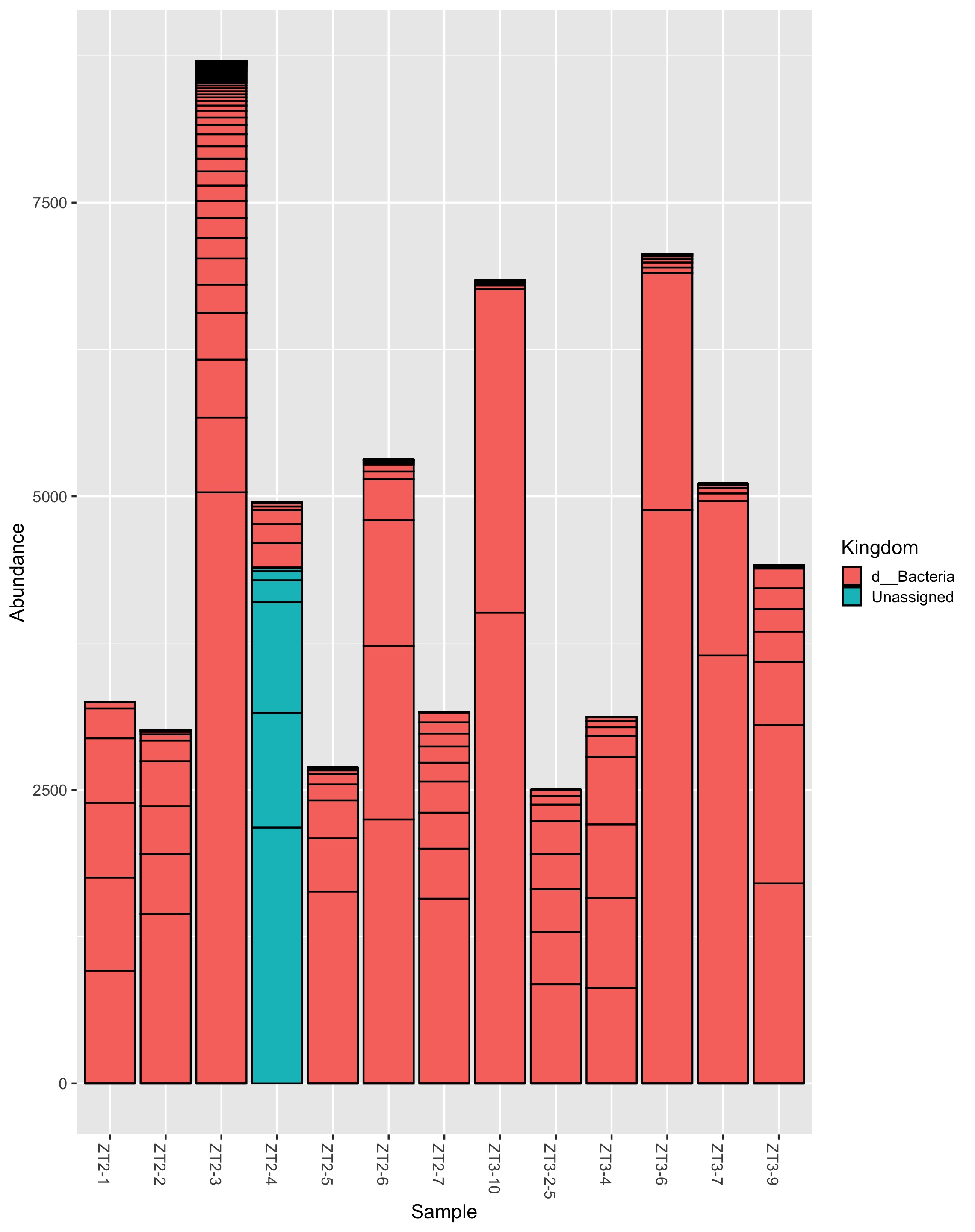

Supplement: Supplementary file 2 — Supplementary file2 (JPG 419 KB) [file 248_2023_2222_MOESM2_ESM.jpg]

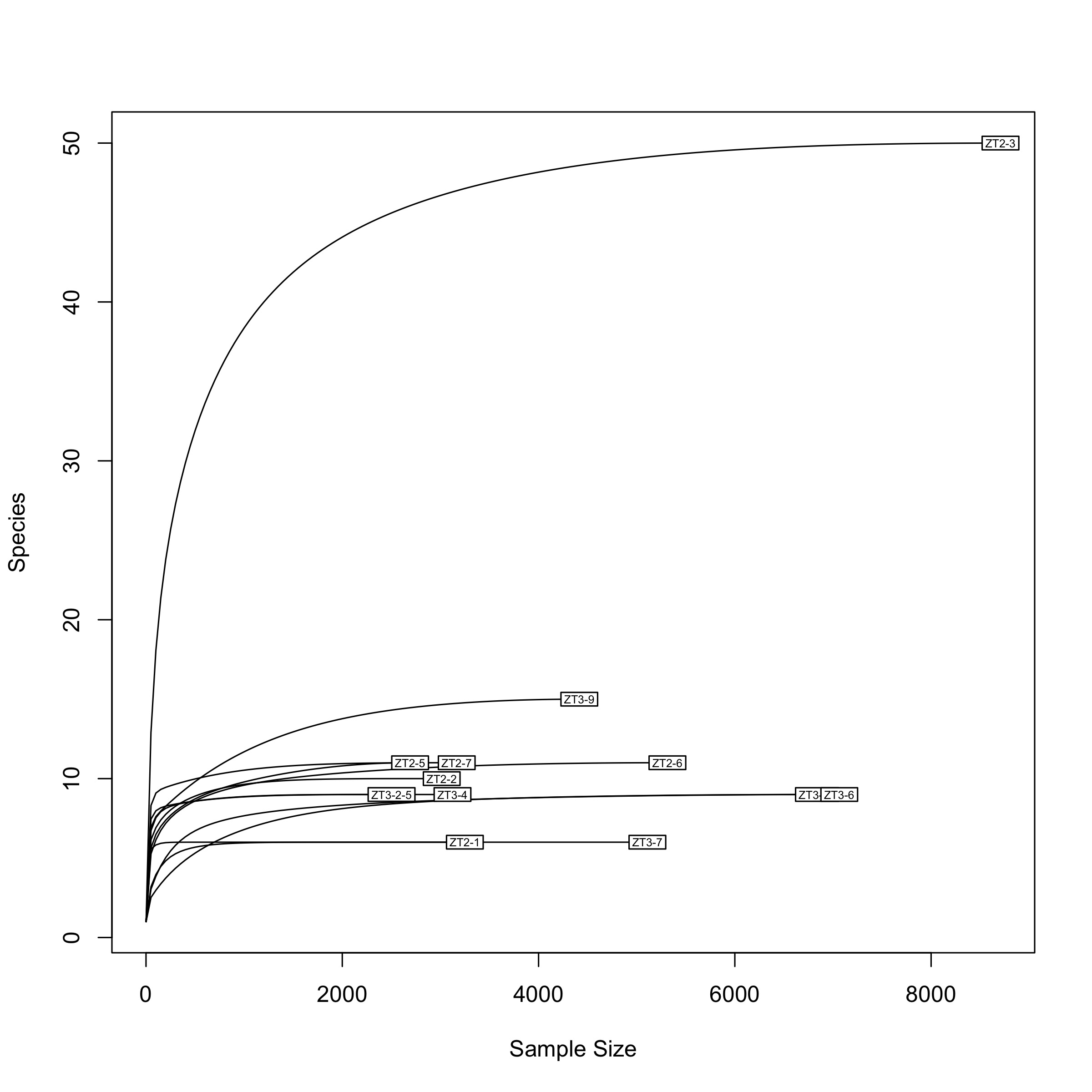

Supplement: Supplementary file 3 — Supplementary file3 (JPEG 211 KB) [file 248_2023_2222_MOESM3_ESM.jpeg]

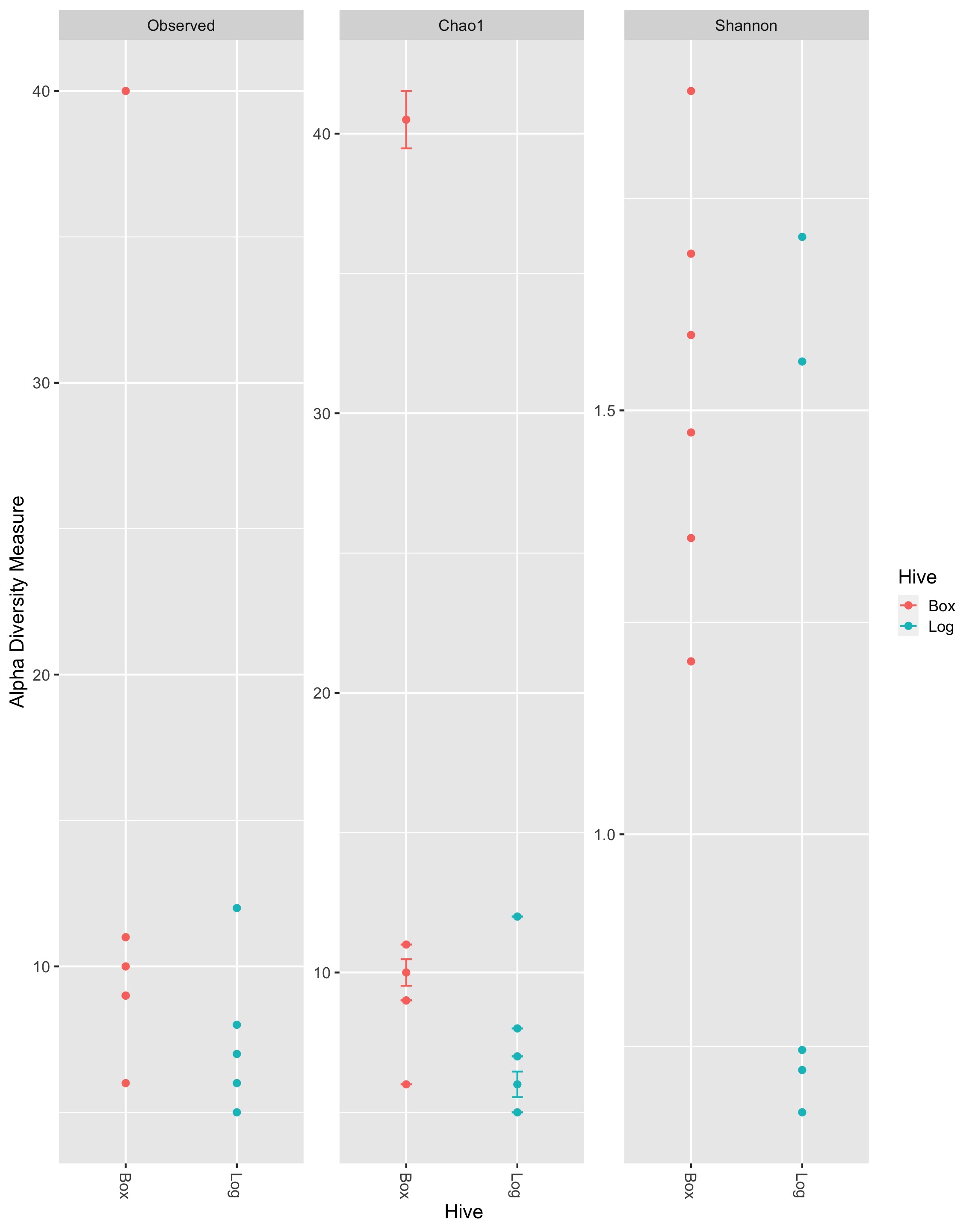

Supplement: Supplementary file 4 — Supplementary file4 (JPG 238 KB) [file 248_2023_2222_MOESM4_ESM.jpg]

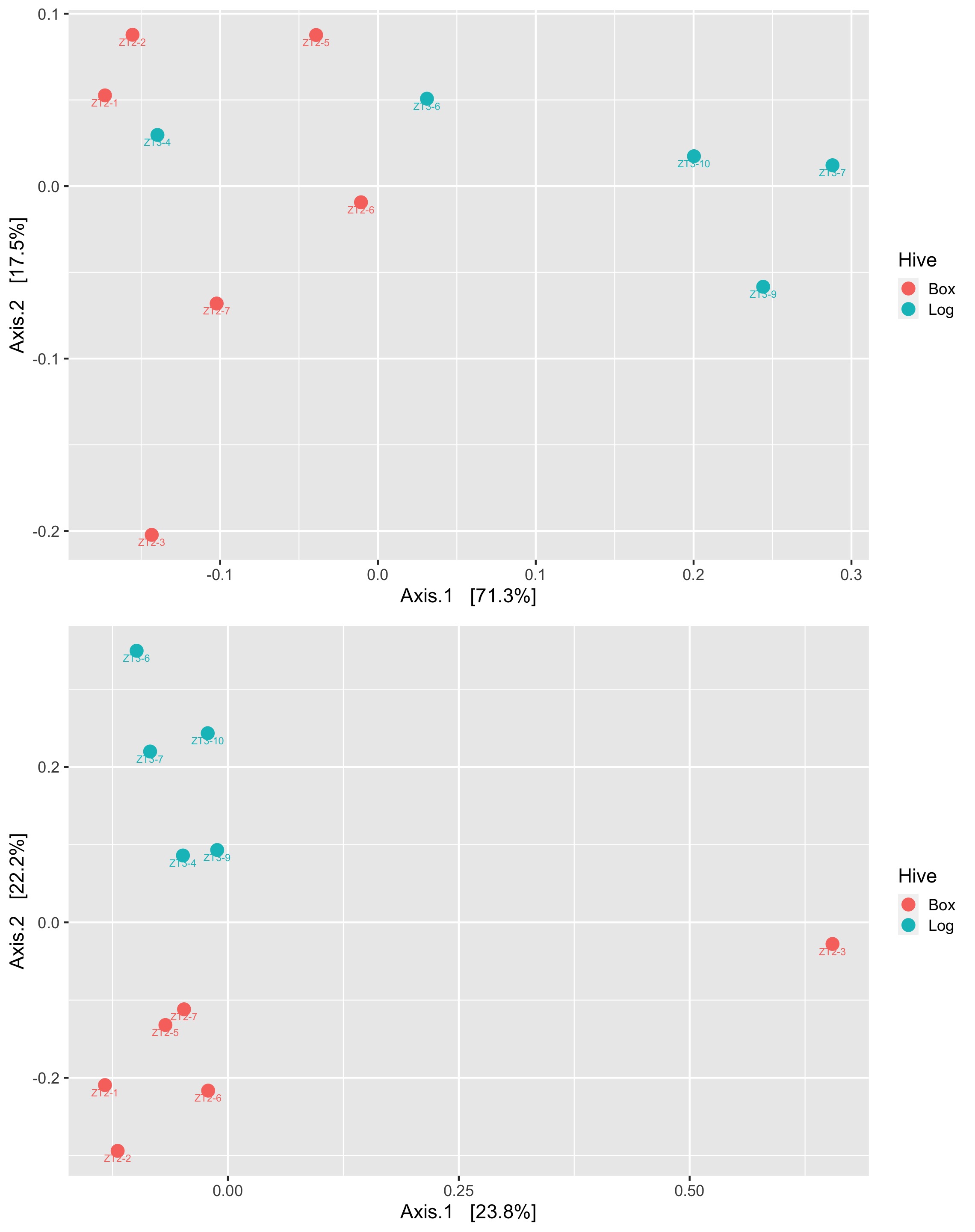

Supplement: Supplementary file 5 — Supplementary file5 (JPG 283 KB) [file 248_2023_2222_MOESM5_ESM.jpg]

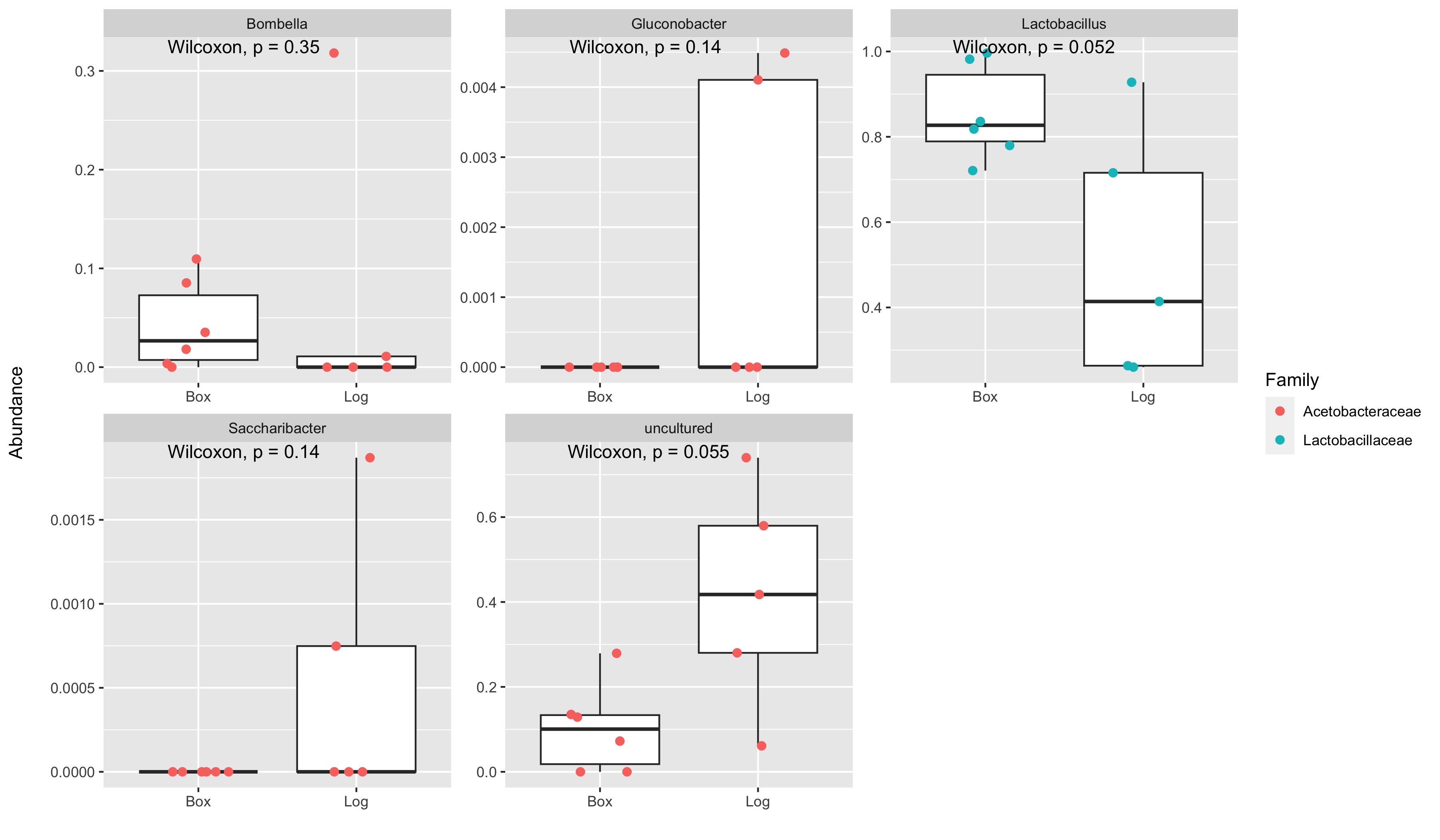

Supplement: Supplementary file 6 — Supplementary file6 (JPEG 389 KB) [file 248_2023_2222_MOESM6_ESM.jpeg]
